# Identification of a Catalytic Active but Non-Aggregating MDM2 RING Domain Variant

**DOI:** 10.1016/j.jmb.2021.166807

**Published:** 2021-03-05

**Authors:** Helge M. Magnussen, Danny T. Huang

**Affiliations:** Cancer Research UK Beatson Institute, Garscube Estate, Switchback Road, Glasgow G61 1BD, United Kingdom; Institute of Cancer Sciences, University of Glasgow, Glasgow G61 1QH, United Kingdom

**Keywords:** Ubiquitin ligase, E3, Protein design, MDM2, Aggregation, GST, Glutathione *S*-transferase, MDM2^f^, *Xenopus tropicalis* MDM2 414-C, MDM2^h^, human MDM2 419-C, MDM2^z^, *Danio rerio* MDM2 407-C, RMSD, Root-mean-square deviation, SPR, Surface plasmon resonance, TEV, Tobacco Etch Virus, E2(UbcH5B)–Ub, isopeptide-linked E2–Ub conjugate, E2(UbcH5B)~Ub, thioester bond-linked E2~Ub conjugate

## Abstract

As a key regulator of the tumour suppressor protein p53, MDM2 is involved in various types of cancer and has thus been an attractive drug target. So far, small molecule design has primarily focussed on the N-terminal p53-binding domain although on-target toxicity effects have been reported. Targeting the catalytic RING domain of MDM2 resembles an alternative approach to drug MDM2 with the idea to prevent MDM2-mediated ubiquitination of p53 while retaining MDM2′s ability to bind p53. The design of RING inhibitors has been limited by the extensive aggregation tendency of the RING domain, making it challenging to undertake co-crystallization attempts with potential inhibitors. Here we compare the purification profiles of the MDM2 RING domain from several species and show that the MDM2 RING domain of other species than human is much less prone to aggregate although the overall structure of the RING domain is conserved. Through sequence comparison and mutagenesis analyses, we identify a single point mutation, G443T, which greatly enhances the dimeric fraction of human MDM2 RING domain during purification. Neither does the mutation alter the structure of the RING domain, nor does it affect E2(UbcH5B)–Ub binding and activity. Hence, MDM2-G443T facilitates studies involving binding partners that would be hampered by the low solubility of the wild-type RING domain. Furthermore, it will be valuable for the development of MDM2 RING inhibitors.

## Highlights

• The MDM2 RING domain has a tendency to form aggregates, which is species dependent.
• The structures of dimeric MDM2 RING domains from frog and fish are presented.
• A G443T substitution strongly reduces aggregation of the human MDM2 RING domain.
• MDM2-G443T exhibits similar structure and activity as wild-type MDM2.

## Graphical abstract

**Figure:**
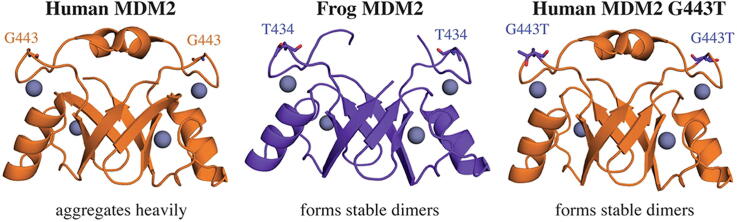

## Introduction

The importance of the ubiquitin ligase murine double mutant 2 (MDM2) as a key negative regulator of the tumour suppressor protein p53 has been studied extensively. MDM2 keeps p53′s activity low under normal conditions, and since p53 induces the expression of MDM2, both proteins are kept at low concentrations.^1^, ^2^ Mouse studies underlined the importance of this feedback loop mechanism: MDM2 knockout is embryonic lethal due to uncontrolled p53 activity levels and can be rescued by simultaneous p53 knockout.^3^, ^4^, ^5^ Upon cellular stress such as DNA damage, p53 is uncoupled from MDM2, allowing it to carry out its anti-tumour functions by inducing the gene expression of proteins involved in DNA repair and apoptosis, depending on the type and degree of stress. p53 knockout mice have a low life expectancy as they lack the ability to respond to DNA damage and are thus highly prone to develop tumours at an early stage. Likewise, p53 mutations that disrupt DNA binding are highly cancerogenic. In fact, half of all human tumours carry a corresponding p53 mutation.^6^ On the other hand, in tumours where p53 is not mutated, MDM2 levels are often found to be elevated, which impairs p53′s activity.^7^

MDM2 regulates p53 by two distinct mechanisms. First, it binds p53 through its N-terminal p53-binding domain, which blocks the transcriptional activity of p53. Second, it promotes the proteasomal degradation of p53 by recruiting Ub conjugating enzyme (E2) thioesterified with ubiquitin (E2~Ub; ~ indicates thioester bond) via its RING domain and mediates ubiquitin (Ub) transfer from E2 to p53,^8^ where Ub is preferentially conjugated to lysine residues located in the C-terminal lysine rich region of p53.^9^ Although the relative importance of these mechanisms is context dependent, *in vivo* mouse studies with ligase defective MDM2 resulted in the same lethality as MDM2 knockout experiments.^10^ The importance of the interplay between MDM2 and p53 is demonstrated by the co-evolution of these proteins, and the high sequence conservation of both MDM2′s p53-binding domain and the RING domain.^11^ Thus, even MDM2 from jawless vertebrates is able to recognize and ubiquitinate human p53 despite 500 million years of evolutionary difference.^12^

MDM2 has been an attractive anti-cancer drug target, especially in tumours where p53 is wild-type with abnormal expression of MDM2.^13^, ^14^ The N-terminal binding interface between MDM2 and p53 was proven to be a promising drug target and the crystal structure for the N-terminal MDM2-p53 complex provided a starting point for structure-guided design of small molecules followed by successful co-crystallization attempts.^15^ A variety of small molecules including imidazolines, oxindoles and benzodiazepines as well as stapled peptides have been developed, which all bind MDM2 in the p53-binding pocket, thereby disrupting p53 binding.^16^ Although advanced derivatives of the imidazoline Nutlin have successfully been tested in clinical trials, negative side-effects have been reported, which are associated with MDM2-mediated off-target ubiquitination and MDM2′s inability to restrict p53′s transcriptional activity.^17^, ^18^

The RING domain of MDM2 has been discussed as an alternative target to stabilize p53.^19^ The rationale for this approach lies in MDM2′s ability to still bind and inhibit p53 when MDM2-mediated ubiquitination is abolished, thereby preventing uncontrolled activation of p53.^8^ The RING domain gains its E3 ligase activity through dimerization with either itself or the RING domain of its catalytic inactive homologue MDMX to form a homodimer or heterodimer, respectively. The dimeric RING domain arrangement is essential for E2~Ub binding and activation as demonstrated by co-crystal structures with UbcH5B–Ub (en dash indicates covalent complex).^8^, ^20^ To date, the development of small molecules that prevent E2~Ub recruitment is still at an early stage. Although 5-Deazaflavin compounds have been introduced as MDM2 RING inhibitors, their potencies are low and optimization is difficult as no structural information is available for how these inhibitors bind MDM2.^19^, ^21^ A key challenge for drug design is the pronounced aggregation tendency of recombinant human homodimeric MDM2 RING domain.^22^ While stable as a GST-fusion protein, human MDM2 RING domain heavily precipitates upon cleavage and forms supramolecular complexes, which makes it a poor candidate for co-crystallization attempts with potential inhibitors.

Here, we show that the aggregation of the MDM2 RING domain is species dependent. By comparing the aggregation behaviour of the MDM2 RING domain from several species followed by systematic alteration of human RING domain sequence, we identify a single point mutation that exclusively yields dimeric protein. Structural and biochemical analyses show that the mutation does not affect E2~Ub binding and the ligase activity. These results identify a suitable strategy for expression and purification of recombinant human MDM2 RING domain for structural studies and should enable future structure-guided design of small molecules that target the RING domain.

## Results

### Aggregation of MDM2 RING domain is species dependent

**Figure 1:**
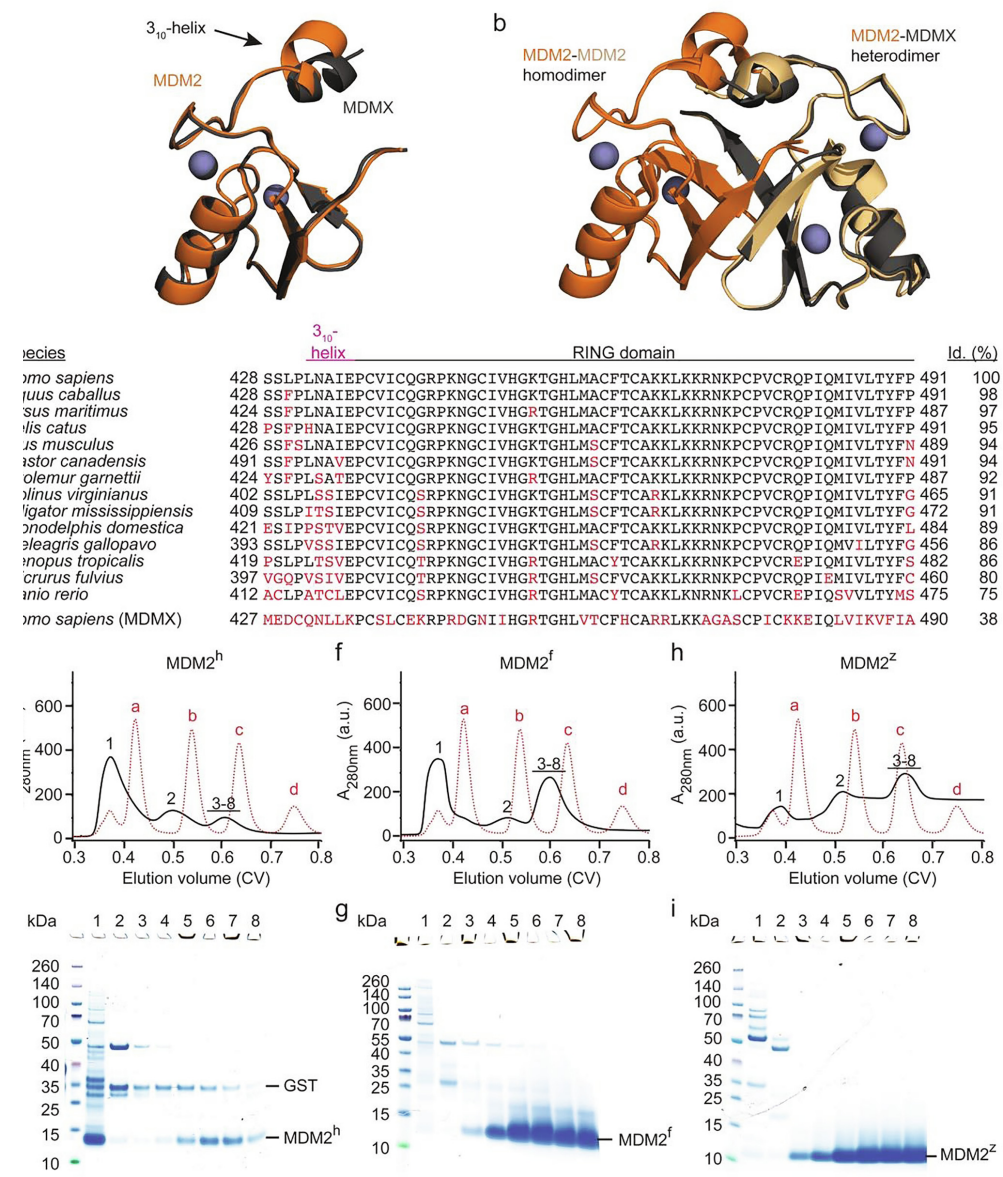
Aggregation of the MDM2 RING domain is species dependent. (a) Superimposition of human MDM2 428-C (orange) and MDMX 428-C (dark-gray) (PDB: 5MNJ). Zinc ions are shown as gray spheres. The 3_10_-helix preceding the RING domain is indicated. (b) Superimposition of the MDM2 RING domain homodimer (PDB: 6SQO; the two monomeric MDM2 RING domains are colored in orange and light orange) and human MDM2-MDMX RING domain heterodimer (PDB: 5MNJ; MDM2 is orange and MDMX is dark-gray). Zinc ions are shown as gray spheres. (c) Sequence alignment of the C-terminal region of MDM2 from different species. The regions corresponding to the 3_10_-helix in MDM2^h^ and the RING domain are indicated. Sequence deviations from human MDM2 are highlighted in red. The sequence identity (id.) compared to human MDM2 is indicated on the right. (d, f, h) Superdex 75 elution profiles of MDM2^h^, MDM2^f^ and MDM2^z^, respectively (shown in black solid line). The elution profile of molecular weight markers (a: bovine serum albumin, 66 kDa; b: carbonic anhydrase, 29 kDa; c: cytochrome C, 12.4 kDa; d: aprotinin, 6,5 kDa) is shown as red dashed line. After removal of GST-tag, the cleaved MDM2 variants (expressed from 24L LB) were applied on a HiLoad 16/600 Superdex 75 column. (e, g, i) SDS-PAGE of indicated fractions from panels d, f, h, respectively. The position of the fractions within the elution profile is indicated by numbers (1–8).

Human MDM2 RING domain is prone to aggregate upon cleavage from a fusion tag^22^ and we observed this behaviour during purification in our prior studies.^8^, ^20^ Despite being predominantly aggregated upon removal of the fusion tag, we found a small fraction that remained as a dimer. Although we were able to purify approximately 1 mg of human MDM2 RING domain from 100-L *E. coli* expression in our prior structural study, this approach offers low reproducibility and is not practical. In contrast, the human MDM2-MDMX RING domain heterodimer predominantly expressed as a dimer and could readily be purified for structural studies.^8^, ^23^ Previously, it was assumed that this difference could be due to structural differences between both dimers. However, our recent crystal structure of the MDM2 homodimer (PDB: 6SQO) showed that the homodimer is structurally very similar to the heterodimer (PDB: 2VJF, RMSD: 0.4 Å).^20^, ^23^ As neither the structural differences between the MDM2 RING domain and MDMX RING domain (Figure 1(a)) nor the structural differences between the homodimer and the heterodimer (Figure 1(a)) could provide an explanation for the homodimer specific aggregation, we wondered whether the aggregation of the homodimer or the stabilization of the heterodimer by MDMX was sequence dependent. The E3 ligase activity of the MDM2 RING domain has been conserved throughout evolution. Important structural features like zinc coordinating residues and the length of the C-terminal tail have been exclusively conserved throughout evolution.^24^ Nevertheless, the sequences of the MDM2 RING domain from different species differ by up to 25 % from human MDM2 (Figure 1(c)). We selected two species, *Xenopus tropicalis* (western clawed frog) and *Danio rerio* (zebrafish), which showed a comparably high sequence variation between themselves and human MDM2. We expressed and purified the MDM2 RING domain from these species and the human counterpart (zebrafish MDM2 407-C, frog MDM2 414-C, and human MDM2 419-C hereafter denoted as MDM2^z^, MDM2^f^, and MDM2^h^, respectively) with an N-terminal cleavable GST-tag and applied the cleaved protein on a size-exclusion chromatography (SEC) column, where protein molecular weight markers were used to estimate whether the cleaved MDM2 RING domain was dimeric (~16 kDa) or aggregated (>66 kDa, the molecular weight of the largest protein molecular weight marker, bovine serum albumin) (Figure 1(d)–(i)). MDM2^h^ precipitated upon removal of the GST-tag and the remaining soluble supernatant mainly eluted in the void volume (~0.35 column volume (CV)), indicating that the protein was heavily aggregated (Figure 1(d) and (e)), whereas only a small fraction eluted at ~ 0.6 CV (estimated molecular weight of 17 kDa; Supplementary Figure 1) consistent of a dimer. Both MDM2^f^ and MDM2^z^ remained stable upon removal of the GST-tag and exclusively eluted as a dimeric protein (Figure 1(f)–(i) and Supplementary Figure 1; estimated molecular weights of 18 and 13 kDa, respectively). Notably, the purified dimeric MDM2^f^ could be concentrated to 12 mg/mL (1.6 mM) without significant precipitation.

### Structural characterization of MDM2^f^ and MDM2^z^

**Figure 2:**
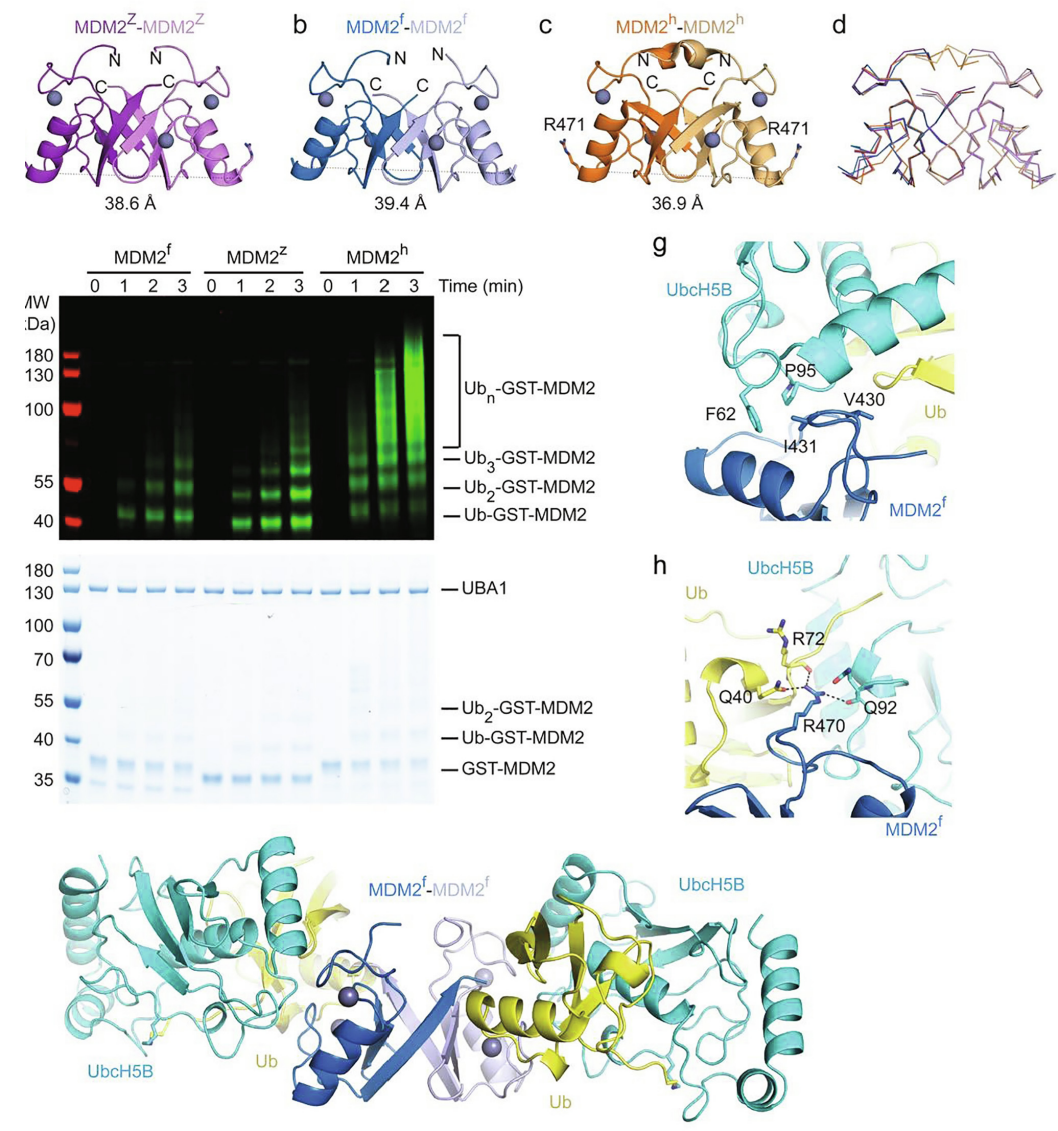
Structural characterization of MDM2^f^ and MDM2^z^. (a–c) Crystal structures of MDM2^z^ (a; purple/light purple), MDM2^f^ (b; blue/light blue) and MDM2^h^ (c; orange/light orange, PDB: 6SQO). Zinc ions are shown as gray spheres. The diameter of the dimer was calculated by measuring the distance between the Cα atoms of R471 (human nomenclature) of both protomers. (d) Superimposition of (a–c) in ribbon form. (e) Reduced SDS-PAGE showing autoubiquitination reactions catalyzed by GST-MDM2 variants using fluorescently-labeled Ub and visualized by an Odyssey CLx Imaging System (top panel) or stained with Coomassie Blue (bottom panel). (f) Crystal structure of the MDM2^f^-UbcH5B–Ub complex. UbcH5B and Ub are colored in cyan and yellow, respectively. MDM2^f^ is colored as in b. (g,h) Close-up views of the key interactions between MDM2^f^ and UbcH5B involving MDM2^f^’s I431 (g) and R470 (h),

We wondered whether the sequence dissimilarity induced structural differences that could account for the different aggregation tendencies of the MDM2 variants and performed protein crystallization for MDM2^f^ and MDM2^z^. We obtained crystals for MDM2^z^ and MDM2^f^, which diffracted to 2.87 and 2.27 Å, respectively (Table 1). The unit cell for the MDM2^z^ crystal contains two molecules of MDM2^z^, which form a dimer. The overall structure is similar to MDM2^h^ (RMSD of 0.6 Å), where all secondary structure elements and the coordination of zinc ions are conserved (Figure 2(a)). There was no electron density for the first 11 residues (407–417, corresponding to 423–433 in MDM2^h^), which are located N-terminal to the RING domain and partly involved in the formation of a 3_10_-helix in MDM2^h^. Likewise, the asymmetric unit of the MDM2^f^ crystal contains two molecules of MDM2^f^, which form a dimer. Similar to MDM2^z^, there was no electron density for residues located N-terminal to the RING domain (414–424, corresponding to 423–433 in MDM2^h^) (Figure 2(b)). In comparison to MDM2^h^, both MDM2^f^ and MDM2^z^ have a slightly larger diameter (Figure 2(a)–(c)). Nonetheless, there is no obvious structural change in the RING domain (Figure 2(d)) that could explain the reduced aggregation observed in MDM2^f^ and MDM2^z^.

**Table 1:** Data collection and refinement statistics

MDM2	MDM2^z^	MDM2^f^	MDM2^f^-UbcH5B–Ub	MDM2^hGT^-UbcH5B–Ub (crystal form 1)	MDM2^hGT^-UbcH5B–Ub (crystal form 2)
**Data collection**					
Space group	*P*2_1_	*P*2_1_	*P*2_1_	*P*6_1_	*P*2_1_2_1_2_1_
Cell dimensions					
*a, b, c* (Å)	23.8, 46.1, 54.2	42.6, 23.7, 55.5	55.0, 153.2, 82.1	129.5, 129.5, 70.8	56.3, 80.7, 135.9
α, β, γ (°)	101.7, 90, 90	90, 101.2, 90	90, 107, 90	90, 90, 120	90, 90, 90
Resolution (Å)	45.21–2.87 (2.92–2.87)^1^	41.75–2.53 (2.60–2.53)	52.62–1.82 (1.85–1.82)	70.75–1.56 (1.59–1.56)	135.91–2.07 (2.11–2.07)
*R_merge_* (%)	27.9 (83.1)	14.4 (73.3)	4.7 (98.9)	12.4 (84.3)	12.7 (73.9)
Completeness (%)	100 (100)	99.8 (100)	98.1 (97.2)	100 (98.3)	100 (99.1)
Multiplicity	3.2 (3.3)	3.1 (3.1)	3.4 (3.4)	17.3 (9.7)	6.4 (6.7)
I/σI	6.3 (2.4)	3.7 (0.8)	13.8 (1.2)	13.8 (2.0)	9.0 (2.0)
CC(1/2)	0.949 (0.667)	0.983 (0.529)	0.998 (0.652)	0.999 (0.518)	0.997 (0.584)
Wilson B (Å^2^)	26.5	45.4	39.8	15.1	27.2
**Refinement**					
Resolution (Å)	53.19–2.87	41.74–2.53	52.62–1.82	64.85–1.56	69.48–2.07
No. reflections	2813	3830	113,660	95,993	38,312
R_work_ (%)	20.7	21.7	19.5	13.4	21.2
R_free_ (%)	28.1	27.9	23.0	18.5	26.6
No. atoms					
Protein	868	822	8803	4620	4472
Water	12	4	356	662	278
Ligand / ion	4	4	9	25	14
B-factors					
Protein	26.35	49.01	53.25	20.63	35.61
Water	13.09	47.24	53.94	36.43	34.63
Ligand / ion	19.92	37.94	39.10	32.48	30.04
RMSD					
Bond lengths (Å)	0.0055	0.0050	0.0085	0.0148	0.0075
Bond angles (°)	1.429	1.377	1.462	1.773	1.473
Ramachandran					
Favoured (%)	95.54	88.99	97.63	97.31	96.41
Outlier (%)	0	0	0	0	0

1 Values in the parentheses are for highest-resolution shell.

**Figure 3:**
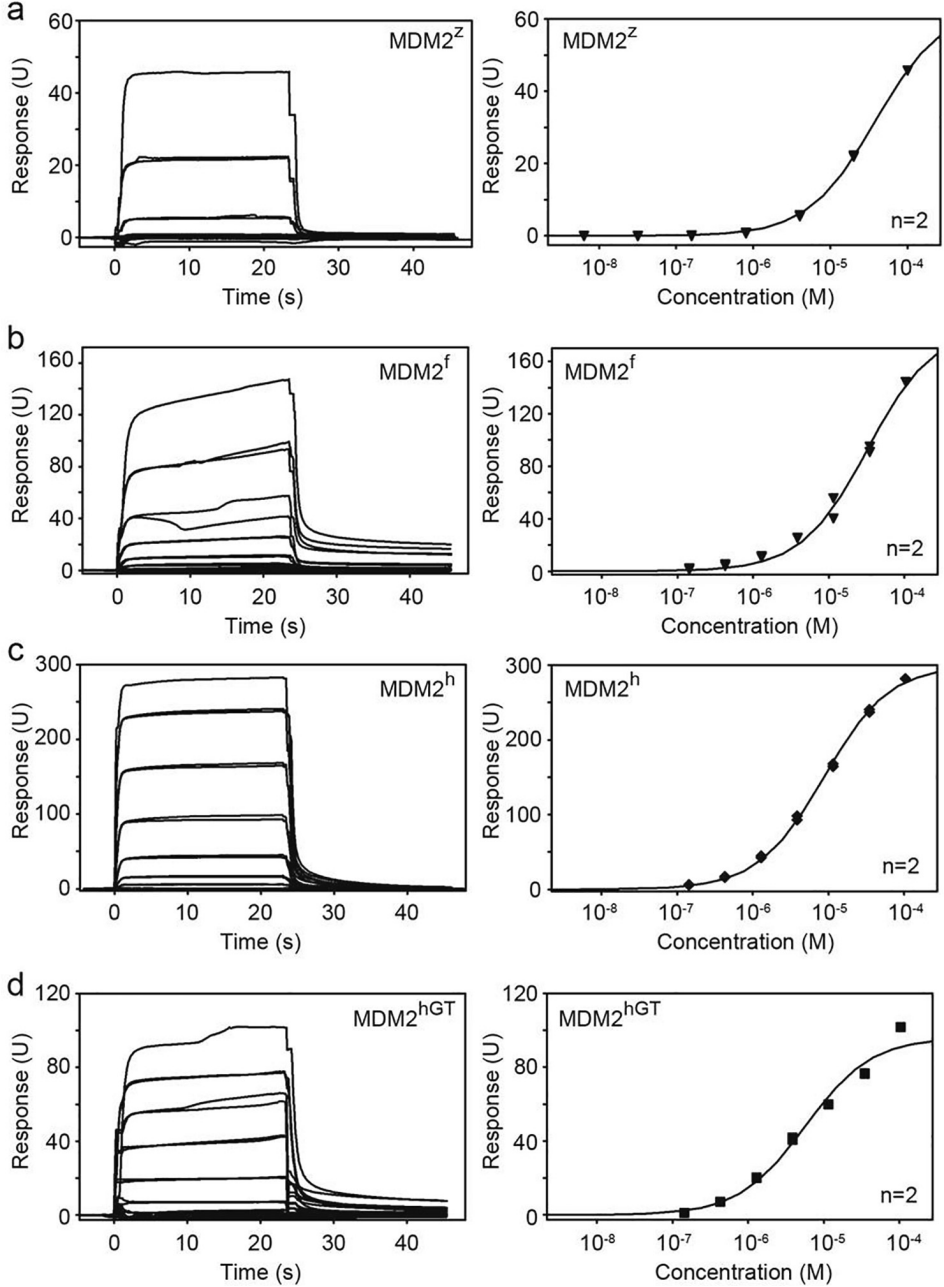
SPR analyses of GST-MDM2 variants and UbcH5B–Ub binding affinities. Representative sensorgrams (left) and binding curves (right) for (a) MDM2^z^ and UbcH5B–Ub, (b) MDM2^f^ and UbcH5B–Ub, (c) MDM2^h^ and UbcH5B–Ub and (d) MDM2^hGT^ and UbcH5B–Ub. n = 2 for each binding curve.

The major difference is the absence of electron density for the 3_10_-helix preceding the RING domain. The 3_10_-helix in MDM2^h^ (residues 432–436) was shown to be important for E2~Ub recruitment. In particular, N433 is involved in a hydrogen bond network with the second MDM2^h^ protomer and the donor Ub.^20^ As there was no electron density for the corresponding residue in MDM2^f^ and MDM2^z^, it is unclear how this would impact on their ability to recruit E2~Ub for catalysis. Before further examination of the relationship between structure, sequence and protein aggregation, we wanted to ensure that both MDM2^f^ and MDM2^z^ are competent E3s. To assess the ability of MDM2^f^ and MDM2^z^ to bind E2~Ub, we performed binding analyses using Surface Plasmon Resonance (SPR). Stable E2–Ub conjugate was obtained by mutating UbcH5B’s catalytic C85 to lysine followed by covalent conjugation of the C-terminus of Ub to the catalytic lysine to form a stable isopeptide linkage that mimics the thioester linkage.^25^ We used the sequence of human UbcH5B, which is identical in western clawed frog, and very similar in zebrafish where it only differs by two residues within the α1-helix that are distal from the MDM2 binding site. MDM2^f^ and MDM2^z^ bind UbcH5B–Ub with a comparable binding affinity, albeit 2.5-fold reduced compared to MDM2^h^ (Figure 3 and Table 2). Next, we performed autoubiquitination assays to verify that MDM2^f^ and MDM2^z^ possess ligase activity. We used GST-tagged proteins to provide lysine residues, since we could not detect autoubiquitination products using cleaved MDM2^h^, presumably due to the lack of acceptor lysine sites for ubiquitination.^20^ Both, MDM2^f^ and MDM2^z^ were competent in generating ubiquitinated products in a time dependent manner and are thus catalytically active (Figure 2(e)). Nevertheless, their activity was reduced compared to MDM2^h^, which could be attributed to the lower binding affinity for UbcH5B–Ub. To elucidate their E2~Ub binding mechanism, we assembled MDM2^f^ in complex with UbcH5B–Ub for crystallization and obtained crystals that diffracted to 1.82 Å (Table 1). The asymmetric unit contains two copies of dimeric MDM2^f^ with each dimer bound to two molecules of UbcH5B–Ub (Figure 2(f)). All UbcH5B–Ub molecules bind MDM2^f^ in a similar manner as observed in the MDM2^h^-UbcH5B–Ub structure,^20^ where key interactions are fully conserved. This includes the hydrophobic interactions between MDM2^f^’s V430 and I431 and UbcH5B’s F62 and P95 and stabilization of the C-terminal tail of Ub by hydrogen bonds initiated by MDM2^f^’s R470 (Figure 2(g) and (h)). Like in the crystal structures of free MDM2^f^, there was only little electron density for residues preceding the RING domain. These structural data suggest that the N-terminal region preceding the RING domain of MDM2^f^ is inherently flexible and the presence of E2~Ub does not restore the 3_10_-helical conformation observed in MDM2^h^. The lack of 3_10_-helices could explain the reduced UbcH5B–Ub binding affinity. Nonetheless, MDM2^f^ binds UbcH5B–Ub in the closed conformation suggesting that it utilizes the same RING E3 mechanism to activate E2~Ub for catalysis.

**Table 2:** Dissociation constants *K*_d_ of MDM2 variants for UbcH5B–Ub. The corresponding sensorgrams and binding curves are shown in Figure 3

Ligand	Analyte	*K*_d_ (μM)
MDM2^f^	UbcH5B–Ub	42.0 ± 0.6
MDM2^z^	UbcH5B–Ub	37.0 ± 1.1
MDM2^h^	UbcH5B–Ub	13.5 ± 0.6
MDM2^hGT^	UbcH5B–Ub	16.3 ± 1.9

### A single point mutation abrogates MDM2 RING domain aggregation

**Figure 4:**
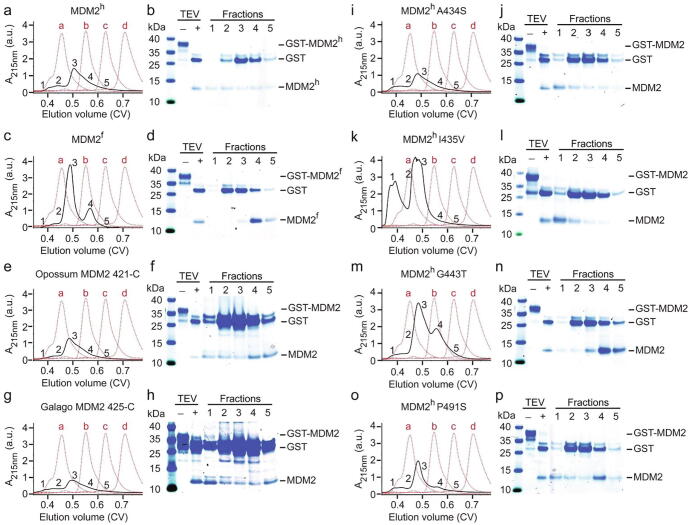
Systematic analysis of MDM2 RING domain aggregation across species (a, c, e, g, i, k, m, o) Superdex 75 elution profiles of MDM2^h^, MDM2^f^, opossum MDM2 421-C, galago MDM2 425-C, MDM2^h^ A434S, MDM2^h^ I435V, MDM2^h^ G443T and MDM2^h^ P491S, respectively (shown as black solid line). The elution profile of molecular weight markers (a: bovine serum albumin, 66 kDa; b: carbonic anhydrase, 29 kDa; c: cytochrome C, 12.4 kDa; d: aprotinin, 6,5 kDa) is shown as red dashed line. GST-MDM2 variants (expressed from 2L LB) were treated with TEV then loaded on a Superdex 75 Increase 10/300 column. (b, d, f, h, j, l, n, p) SDS-PAGE showing the GST-MDM2 variants before and after TEV treatment to release the GST-tag (labeled ‘–’ and ‘+’, respectively) and single fractions of the corresponding SEC experiments from panels a, c, e, g, i, k, m, o, respectively. The position of the fractions within the elution profile is indicated by numbers (1–5).

It is unclear to which extent the disordered N-terminal region preceding their RING domains or sequence variations in the RING domain contribute to the reduced aggregation. In order to get a better understanding of MDM2 RING domain aggregation, we selected a few species that were closer to MDM2^h^ than MDM2^z^ and MDM2^f^ (Figure 1(c)). GST-tagged MDM2 RING domain was incubated with TEV protease to cleave the GST-tag and the cleaved products were then applied on an analytical SEC column to assess the aggregation state of the MDM2 RING domain. *Monodelphis domestica* (opossum) MDM2 RING domain eluted mainly at ~ 0.6 CV consistent of a dimer, and only a small fraction eluted as aggregate. In contrast, a substantial fraction of *Otolemur galettii* (galago) MDM2 RING domain aggregated (Figure 4(a)–(h)). When we compared their sequences against MDM2^h^, MDM2^f^ and MDM2^z^, we found that only A434, I435, G443 and P491 were unique to galago MDM2 and MDM2^h^. A434 and I435 are located within the N-terminal region preceding the RING domain, whereas G443 and P491 are located within the RING domain. We hypothesized that these residues might promote aggregation and converted MDM2^h^ into MDM2^f^ by introducing single point mutations, where we replaced these residues with the corresponding residues of MDM2^f^ and analyzed their aggregation state on an analytical SEC column (Figure 4(i)–(p)). MDM2^h^ A434S, MDM2^h^ I435V and MDM2^h^ P491S behaved like wild-type MDM2^h^, where removal of the GST-tag caused precipitation and the SEC elution profiles of the soluble supernatant were comparable to wild-type MDM2^h^, In contrast, MDM2^h^ G443T (hereafter referred to as MDM2^hGT^) was stable upon removal of the GST-tag and the SEC elution profile showed reduced aggregation with bulk of MDM2^hGT^ eluting at ~0.6 CV consistent of a dimer.

### Structural and functional characterization of MDM2^hGT^

**Figure 5:**
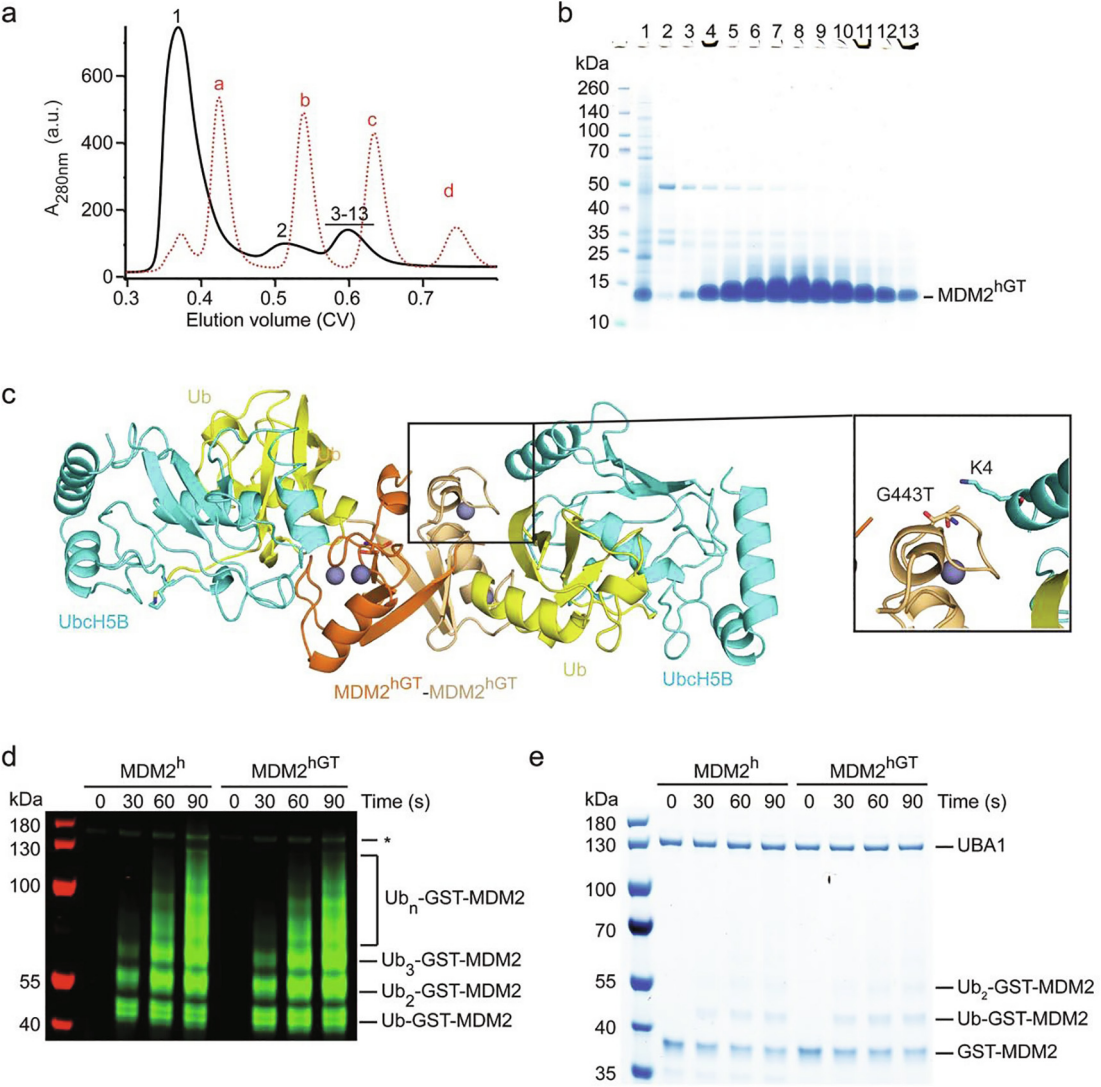
Functional and structural characterization of MDM2^hGT^. (a) Superdex 75 elution profile of MDM2^hGT^ from a large-scale purification (shown as black solid line). The elution profile of molecular weight markers (a: bovine serum albumin, 66 kDa;b: carbonic anhydrase, 29 kDa; c: cytochrome C, 12.4 kDa; d: aprotinin, 6,5 kDa) is shown as red dashed line. (b) SDS-PAGE showing the purity of single fractions from a. The position of the fractions within the elution profile is indicated by numbers (1–13). The large absorbance in fraction 1 is due to the presence of other contaminants. (c) Crystal structure of the MDM2^hGT^-UbcH5B–Ub complex. The two MDM2^hGT^ monomers are colored in orange and light orange. Zinc ions are shown as gray spheres. UbcH5B and Ub are colored in cyan and yellow, respectively. A representative close-up view of the local environment of G443T including the sidechain of UbcH5B’s K4 are shown. (d,e) Reduced SDS-PAGE showing autoubiquitination reactions catalyzed by GST-MDM2^h^ and GST-MDM2^hGT^ using fluorescently-labeled Ub and visualized by an Odyssey CLx Imaging System (d) or stained with Coomassie Blue (e). Asterisk in d indicates non-reducible E1–Ub product.

MDM2^h^’s G443, or the corresponding residue in MDM2^f^, T434, is solvent exposed and located within a loop. None of the available crystal structures could explain why a glycine at this position would cause the MDM2 RING domain to aggregate and that a threonine would prevent it. The corresponding residue in MDM2 from zebrafish and opossum, which both predominantly form stable dimers, is serine, suggesting that the polar side chain at this site could reduce aggregation. Since the G443T substitution produced a stable MDM2^h^ dimer, we wanted to understand its impact on MDM2^h^’s structure and function. We purified MDM2^hGT^ on a large scale with the aim to crystallize it in complex with UbcH5B–Ub. The SEC elution profile (Figure 5(a) and (b)) and the yield were comparable to the purification of MDM2^f^ (Figure 1(f) and (g)). We obtained diffracting crystals in two different forms (Table1), of which one is isomorphous to the crystal form of the MDM2^h^-UbcH5B–Ub complex (PDB: 6SQO). Both unit cells contain a single MDM2^hGT^ dimer bound to two UbcH5B–Ub molecules (Figure 5(c)). The structure of MDM2^hGT^ and the orientation of UbcH5B–Ub are indistinguishable from the structure of wild-type MDM2^h^ bound to UbcH5B–Ub, indicating that the point mutation did not affect the ability of the RING domain to recruit UbcH5B–Ub. G443T is located near the UbcH5B binding site and is in proximity to UbcH5B’s K4. However, in all MDM2^hGT^-UbcH5B–Ub complexes in the asymmetric units of both datasets, the G443T sidechain is not within hydrogen bond distance of UbcH5B’s K4 and in one instance UbcH5B’s K4 sidechain could not be modelled due to poor electron density. Based on these observations, the G443T substitution impacts neither the RING domain fold nor UbcH5B–Ub binding. Indeed, SPR binding analysis showed that the G443T substitution had minimal effect on UbcH5B–Ub binding (Figure 3(d) and Table 2). Consequently, the autoubiquitination activity of MDM2^hGT^ is comparable to the wild-type MDM2^h^ (Figure 5(d) and (e)). These results demonstrate that the G443T variant is fully functional and exhibits a similar RING E3 property as MDM2^h^.

## Discussion

p53 and its main negative regulator MDM2 have co-evolved since their first appearance in single cell organisms over a billion years ago.^11^ In MDM2, both the p53-binding domain and the RING domain show a high degree of sequence conservation and are crucial for p53 regulation. The structural characterization of the p53-binding domain of MDM2 in complex with p53 peptide has paved the way for the development of small molecules and stapled peptides that are currently in clinical trial to reactivate p53 activity in cancer patients.^26^ In contrast, structural analysis of MDM2 RING domain remains challenging as recombinant human MDM2 RING domain predominantly aggregates. Here, we show that MDM2 RING domain aggregation is species specific. By comparing the structures and sequences of MDM2 from different species in combination with mutagenesis analyses, we identify a single substitution G443T that stabilizes MDM2^h^ predominantly in the dimeric state. Biochemical and structural analyses show that MDM2^hGT^ does not alter MDM2 RING domain structure and exhibits similar E2~Ub binding affinity and activity as MDM2^h^. These findings demonstrate that MDM2^hGT^ can serve as a valuable tool for future structural characterization of potential binding partners of MDM2 RING domain and aid the development of small molecule inhibitors of MDM2 ligase activity.

**Figure 6:**
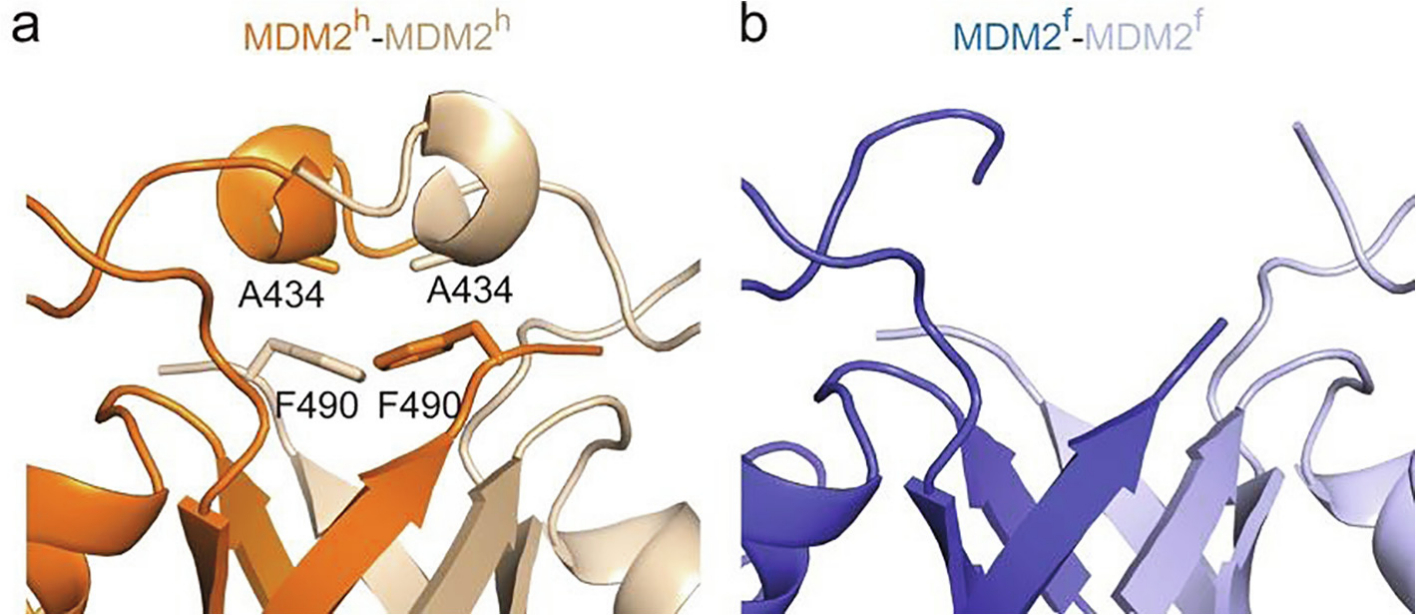
3_10_-helix precedes the RING domain in MDM2. (a) Close-up view of the 3_10_-helix that precedes the RING domain in MDM2^h^. Location of A434 within MDM2^h^ is indicated. (b) Close-up view of the corresponding region in MDM2^f^. No electron density was observed in this region.

Ancestral MDM2 was shown to bind and ubiquitinate human p53 in cells.^12^ Here, we report crystal structures of MDM2 RING domain from two different species, western clawed frog and zebrafish. The fold of the RING domain and its ability to recruit UbcH5B–Ub in the closed conformation are conserved; supporting the hypothesis that MDM2′s function as a ubiquitin ligase has been conserved from an evolutionary point of view. However, the binding affinity is reduced compared to the human counterpart. A possible explanation for this observation could be the sequence difference for residues preceding the RING domain, which form a 3_10_-helix in MDM2^h^ but lack electron density in all MDM2^f^ and MDM2^z^ structures reported in this study. The helices are stabilized by a tight hydrophobic packing arrangement involving residues A434 and F490 of each protomer (Figure 6(a)). In MDM2^f^ and MDM2^z^, A434 is replaced with serine and cysteine, respectively (Figure 1(c)). Both serine and cysteine would likely cause a steric clash and their polar side chains would disrupt the hydrophobic packing, thereby precluding a similar helical arrangement (Figure 6(b)). The 3_10_-helices in MDM2^h^ contribute to the stabilization of UbcH5B–Ub in the closed conformation and their absence in MDM2^f^ and MDM2^z^ might be responsible for the reduced binding affinity for UbcH5B–Ub. Although early vertebrate MDM2 RING domain was an active ubiquitin ligase, the 3_10_-helices might have evolved to enhance MDM2′s ligase activity in placental mammals where A434 is conserved (Figure 1(c)). In other animal classes, it is replaced with serine, threonine, isoleucine or cysteine (Figure 1(c)), which will likely disrupt the hydrophobic packing due to bulky or polar side chains. What might the consequences of this structural dissimilarity be? The residues N-terminal to the RING domain were suggested to be important for XIAP IRES mRNA binding,^27^ which could thus be a species dependent MDM2 feature. The absence of the 3_10_-helices might also affect MDM2′s posttranslational regulation. We showed that DNA-damage-induced phosphorylation of S429 enhances the autoubiquitination activity of MDM2^h^, and this effect was abolished by a helix-disrupting mutation (A434R).^20^ Species lacking the 3_10_-helices potentially respond differently to S429-phosphoregulation compared to MDM2^h^. Various phosphorylation sites near the RING domain have been identified^28^ and a serine at position 434 could not only be responsible for the disruption of the 3_10_-helices but might also serve as a phosphorylation site itself and thereby alter E2~Ub recruitment.

We identified G443 as a single residue being responsible for the pronounced aggregation of MDM2^h^, which could be eliminated by a single point mutation G443T. As the structures of MDM2^h^ and MDM2^hGT^ are indistinguishable, it remains unclear why the wild-type aggregates. It is noteworthy that in the human MDM2-MDMX heterodimer G443 is present in MDM2 but we did not observe aggregation, suggesting that the symmetric nature of MDM2^h^ homodimer might contribute to the initiation of this process. The formation of supramolecular assemblies has been observed for other RING E3s,^29^ but only little is known about the molecular mechanism of their formation. DNA damage-induced phosphorylation of MDM2 was shown to reduce the size of MDM2 oligomers suggesting that while the RING domain has a tendency to oligomerize, post-translational modification at other regions of MDM2 could regulate its oligomeric state.^28^ It would be interesting to know whether the G443T mutation changes the oligomeric state of MDM2 in cells and how this affects its function. It is worthwhile to note that G443 is only present in placental mammals and might thus be an evolutionary fine-tuning tool to regulate MDM2.

## Methods

### Protein purification

MDM2 constructs were cloned into pGEX-4 T1 and expressed with an N-terminal GST-tag followed by a TEV cleavage site, human UBA1 was cloned into pET21d with an N-terminal 6xHis-tag,^30^ UbcH5B was cloned into pRSFDuet-1 and Ub was cloned into pRSFDuet-1 with an N-terminal 6xHis-tag followed by a TEV cleavage site and a GGS linker to enhance the cleavage efficiency.^20^ All UbcH5B proteins in this study contained the S22R mutation to block backside binding of Ub.^31^ For UbcH5B–Ub conjugates used for crystallization and SPR binding analyses, the catalytic cysteine was mutated to lysine (C85K) to obtain a stable UbcH5B–Ub conjugate with isopeptide-linked Ub.^32^ All proteins were expressed in *Escherichia coli* BL21(DE3) GOLD. Cells were grown in LB medium to OD_600_ = 0.6–1.0 at 37 °C, induced with 0.2 mM Isopropyl-β-D-1-thiogalactopyranoside and protein expression was conducted for 16–20 h at 20 °C. Cells were resuspended and lysed in the corresponding wash buffer of the first purification step supplemented with 2.5 mM phenylmethylsulfonyl fluoride. UBA1, UbcH5B, Ub and MDM2 were purified as described previously.^20^, ^25^ UbcH5B–Ub and fluorescently labeled Ub were generated as described previously.^20^, ^32^ For SPR and ubiquitination assays, GST-MDM2 variants were purified by glutathione Sepharose affinity chromatography followed by gel filtration chromatography. For crystallization, GST-MDM2 variants were treated with TEV to release the GST-tag and the cleaved MDM2 variants were subjected to HiLoad 16/600 Superdex 75 gel filtration chromatography (GE Healthcare). The oligomeric state of the MDM2 variants was assessed by comparison with molecular weight markers (Sigma-Aldrich). All MDM2 variants were stored in buffer containing 25 mM Tris-HCl, pH 7.6, 0.4 M NaCl and 1 mM DTT. Protein concentrations were determined by absorbance at 280 nm for Ub and Bio-Rad protein assay with BSA as a standard for other proteins.

### Crystallization

Crystals were grown at 19 °C using sitting drop vapour diffusion technique (drop volume: 0.4 μL) and where required optimized by hanging drop vapour diffusion techniques (drop volume: 2.0 μL). All proteins were mixed with reservoir solution at 1:1 ratio.

MDM2^z^ (3 mg/mL) crystals were obtained in condition containing 0.1 M imidazole, pH 6.5, 0.12 M monosaccharides, 37.5 % (w/v) MPD_P1K_P3350 (Morpheus, Molecular Dimensions) and flash-frozen in the same condition.

MDM2^f^ (5–9 mg/mL) crystals were obtained in condition containing 0.1 M PCTP, pH 7.0, 25 % (w/v) PEG 1500 (PACT premier, Molecular Dimensions) and flash-frozen in the same condition containing 30 % (v/v) MPD.

MDM2^f^-UbcH5B–Ub crystals were obtained by mixing MDM2^f^ (5–9 mg/mL) and UbcH5B–Ub (15 mg/mL) at 1:1 molar ratio in condition containing 0.1 M HEPES, pH 7.0, 10 % (w/v) PEG 20,000 and flash-frozen in the same condition containing 25 % (v/v) ethylene glycol.

MDM2^hGT^-UbcH5B–Ub crystals were obtained by mixing MDM2^hGT^ (11 mg/mL) and UbcH5B–Ub (15 mg/m) at 1:1 ratio. Crystal form 1 was obtained in condition containing 0.1 M Tris, pH 8.0, 0.075 M NaOAc, 0.1 M NaCl, 15 % (w/v) PEG Smear Medium and flash-frozen in the same condition containing 25 % (v/v) ethylene glycol. Crystal form 2 was obtained in condition containing 0.1 M HEPES, pH 7.5, 0.2 M (NH_4_)NO_3_, 20 % (w/v) PEG Smear Broad. Both crystal forms were flash-frozen in the same condition containing 25 % (v/v) ethylene glycol.

### Structure determination

Diffraction data were collected at beamlines I03, I04 and I04-1, Diamond Light Source (DLS). Data were processed by automated XDS^33^ and reduced with fast_DP (MDM2^f^-UbcH5B–Ub), autoPROC (MDM2^z^)^34^ or Xia2 package (all other datasets).^35^ The structures were solved by molecular replacement using PHASER,^36^ followed by consecutive rounds of refinement with REFMAC5^37^ and model building with COOT.^38^ Refinement statistics are shown in Table 1.

### SPR analysis

SPR binding experiments were performed at 25 °C on a Biacore T200 instrument using a CM-5 chip (GE Healthcare) with coupled anti-GST antibody as described previously.^20^ Briefly, GST-tagged MDM2 variants were coupled on the chip and a serial dilution of UbcH5B–Ub in running buffer containing 25 mM Tris-HCl, pH 7.6, 150 mM NaCl, 1 mM DTT and 0.005% (v/v) Tween-20 was used as analyte. Two technical replicates were performed and data were analyzed with BIAevalution (GE Healthcare) and Scrubber2 (BioLogic Software).

### *In vitro* autoubiquitination assay

UbcH5B (5 μM) was pre-charged for 20 min with UBA1 (0.2 μM) and fluorescently-labeled Ub (70 μM) in 50 mM Tris, pH 7.6, 50 mM NaCl, 5 mM MgCl_2_, 5 mM ATP at 23 °C. The reaction was started by adding GST-MDM2 variants (5 μM; in 50 mM Tris, pH 7.6, 400 mM NaCl) and stopped at the indicated time point with 4X LDS Loading dye containing 400 mM DTT followed by separation on SDS-PAGE. The ubiquitinated products were visualized with an Odyssey CLx Imaging System (LI-COR Biosciences) and staining with coomassie. Parentheses indicated final concentration of proteins in the reaction.

### Analytical size-exclusion chromatography

GST-tagged MDM2 variants were concentrated to 3 mg/mL and incubated with 1:10 TEV protease for 16–20 h at 4 °C to cleavage the GST-tag. Cleavage was confirmed by SDS-PAGE. The soluble fraction of the cleaved products was applied on a Superdex 75 Increase 10/300 SEC column (GE Healthcare) and the elution profiles were analyzed by comparison with molecular weight markers (Sigma-Aldrich) and by SDS-PAGE.

## Accession numbers

PDB: 7AH2, 7AHY, 7AHZ, 7AI0 and 7AI1

## Data availability

Atomic coordinates and structure factors are deposited in the Protein Data Bank with ascension codes 7AH2 (MDM2^z^), 7AHY (MDM2^f^), 7AHZ (MDM2^f^-UbcH5B–Ub), 7AI0 (MDM2^hGT^-UbcH5B–Ub, crystal form 1), 7AI1 (MDM2^hGT^-UbcH5B–Ub, crystal form 2).

## CRediT authorship contribution statement

**Helge M. Magnussen:** Conceptualization, Methodology, Investigation. **Danny T. Huang:** Conceptualization, Supervision, Funding acquisition.
