# Supplementary material for: Identification of a Catalytic Active but Non-Aggregating MDM2 RING Domain Variant
Source: J Mol Biol. 2021 Mar 5;433(5):166807. doi: 10.1016/j.jmb.2021.166807 (PMC7895813; doi:10.1016/j.jmb.2021.166807)
Supplement: Supplementary data 1 [file mmc1.pdf]

Supplementary Information

Identification of a catalytic active but non-aggregating MDM2 RING domain variant

Helge M. Magnussen and Danny T. Huang

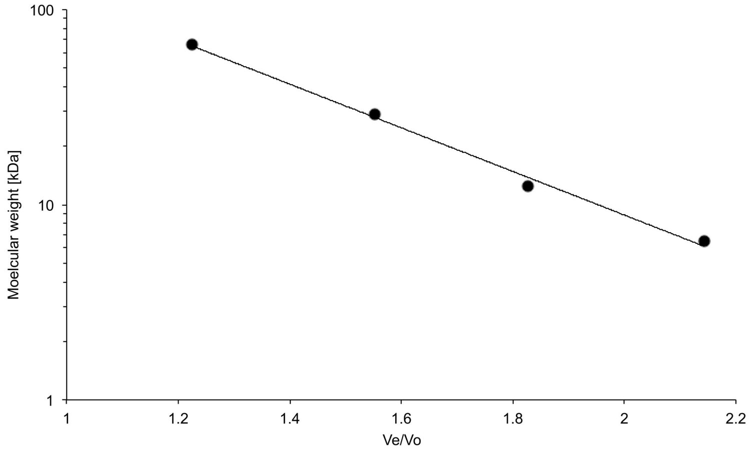

Supplementary Figure 1. A semi-log plot of molecular weights of protein standard versus elution volume (Ve)/void volume (Vo), related to Figure 1d–i.
